# α-Synuclein in traumatic and vascular diseases of the central nervous system

**DOI:** 10.18632/aging.103675

**Published:** 2020-11-07

**Authors:** Hong Zeng, Nan Liu, Xiao-Xie Liu, Yan-Yan Yang, Mou-Wang Zhou

**Affiliations:** 1 Department of Rehabilitation Medicine, Peking University Third Hospital, Beijing 100191, China

**Keywords:** α-Synuclein, spinal cord injury, brain injury, ischemic stroke, posttranslational modification

## Abstract

α-Synuclein (α-Syn) is a small, soluble, disordered protein that is widely expressed in the nervous system. Although its physiological functions are not yet fully understood, it is mainly involved in synaptic vesicle transport, neurotransmitter synthesis and release, cell membrane homeostasis, lipid synthesis, mitochondrial and lysosomal activities, and heavy metal removal. The complex and inconsistent pathological manifestations of α-Syn are attributed to its structural instability, mutational complexity, misfolding, and diverse posttranslational modifications. These effects trigger mitochondrial dysfunction, oxidative stress, and neuroinflammatory responses, resulting in neuronal death and neurodegeneration. Several recent studies have discovered the pathogenic roles of α-Syn in traumatic and vascular central nervous system diseases, such as traumatic spinal cord injury, brain injury, and stroke, and in aggravating the processes of neurodegeneration. This review aims to highlight the structural and pathophysiological changes in α-Syn and its mechanism of action in traumatic and vascular diseases of the central nervous system.

## Structure of α-Synuclein

α-Syn is composed of 140 amino acids. Snca encodes α-Syn, and wild-type (WT) α-Syn is an inherently disordered protein (IDP). The gene encoding the protein is located on human chromosome 4 and occupies a region of approximately 114 kb in the genome. α-Syn accounts for 1% of the total protein content of neurons and has a predominantly presynaptic localization. It is widely distributed within neurons and is found in the cytoplasm, nucleus, mitochondria, and mitochondria-associated membranes. In the cytoplasm, one-third of the protein binds to the synaptic membrane [1–3]. α-Syn has no persistent structure under physiological conditions and is mainly monolithic and inherently disordered.

**Figure 1:**
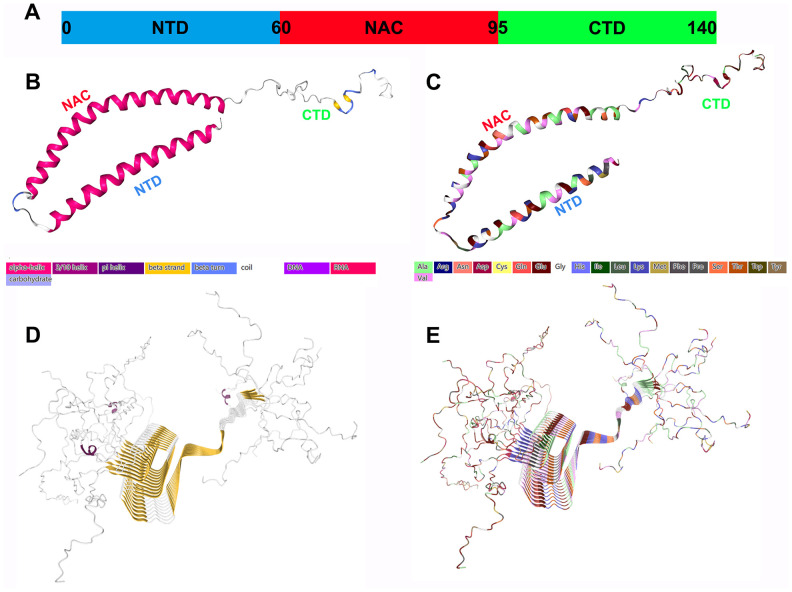
**Structural characteristics of α-Syn monomers and fibril aggregates.** (**A**) α-Syn is composed of three different regions: a positively charged amphiphilic N-terminus (residues 1-60), a hydrophobic nonamyloid (NAC) region (residues 61-95), and a negatively charged CTD (residues 96-140); they may have different functions. (**B**) The secondary structure of α-Syn. (**C**) The structure of the amino acid residues of α-Syn. (**D**) The fibril structure (secondary structure) of α-Syn. (**E**) The fibril structure (amino acid residues) of α-Syn. All the structure diagrams are from the PDB database (https://www.rcsb.org/), and the corresponding colors are marked.

Under physiological conditions, α-Syn displays a disordered structure *in vitro*. Its sequence is usually divided into the following three main regions (Figure 1):

The N-terminal amphipathic domain (residues 1–60): The amphiphilic N-terminal domain (NTD) is composed of six imperfect repeats (KTKEGV) with which the protein interacts with lipids;

The nonamyloid β component (NAC) region (residues 61-95): This is a central hydrophobic region with oligomeric properties that participates in α-Syn protein aggregation and appears to be essential for α-Syn fibril formation. It integrates with the highly acidic C-terminal domain (CTD). Since these interactions involve electrostatic properties, the ionic strength or pH of the solution can strongly increase the aggregation of α-Syn [4];

The CTD (residues 96-140): This is a highly acidic and proline-rich region with no obvious structural tendency. The negative charge in the acidic portion of the CTD, confers chaperone-like properties and can inhibit the oligomerization process, which is essential for preventing rapid α-Syn fibrillation [5].

Natural α-Syn is usually an IDP or unstructured monomer, and the disordered monomer form is the main state of α-Syn in aqueous solution. The α-Syn tetramer is another form with a stable helix structure that coexists with the monomer in the cell [6]. The use of denaturing detergents and cell lysis methods may destabilize the tetramers, thereby forming monomers. Tetramer can maintain α-Syn steady state and resist aggregation [7]. Therefore, α-Syn naturally exists as a helical tetramer, and it is in a dynamic equilibrium state with disordered monomers, which is the overall dominant state. Missense mutations in the α-Syn gene reduce the ratio of the tetramer to the monomer, which promotes the transition to a disease state.

## The Physiological Function of α-Syn

In nondisease states, the function of α-Syn is still poorly understood, although increasing evidence suggests that the protein mediates the transport of vesicle axons, regulates neurotransmitter release, and mediates the interaction and assembly of synaptic vesicles. It also undergoes strong binding to negatively charged vesicles *in vitro* and has the function of inhibiting membrane fusion [8]. The physiological effects of neurons rich in α-Syn expression include maintenance of mitochondrial morphology, regulation of transport, and clearance of substances. Recent reports indicate that α-Syn may benefit to improve neuronal microtubule dynamics and lymphocyte development [9–10]. In short, its biological function is inseparable from the biological significance of cell membrane homeostasis and synaptic regulation. Therefore, vesicles composed of mixed lipids, which mimic lipids in neuronal cells, should be studied to discover the true biological significance of α-Syn *in vivo* and *in vitro*.

## α-Syn participates in synaptic vesicle transport and neurotransmitter synthesis and release

In rodents, α-Syn expression is detected shortly after birth, continues to increase until one month of age, and then reaches a stable level that persists into adulthood. Similarly, in cultured rat neurons, synaptic development preceded the expression of α-Syn and translocation to the axonal end [11]. α-Syn is an intracellular protein, and under physiological conditions, the level of α-Syn in the central nervous system (CNS) is quite high. However, it has been suggested that a large proportion of α-Syn may be extracellular and may be secreted and transmitted between neuronal cells [12]. α-Syn is present in biological fluids, such as cerebrospinal fluid (CSF) and plasma, in patients and normal subjects, which is also evidence of α-Syn release. α-Syn is also found in peripheral neurons, hematopoietic cells in the bone marrow, and circulating blood cells, including red blood cells (RBCs), platelets, and lymphocytes; this distribution depends on α-Syn transport between cells, which occurs via exosomes and other extracellular vesicles (EVs) [13].

**Figure 2:**
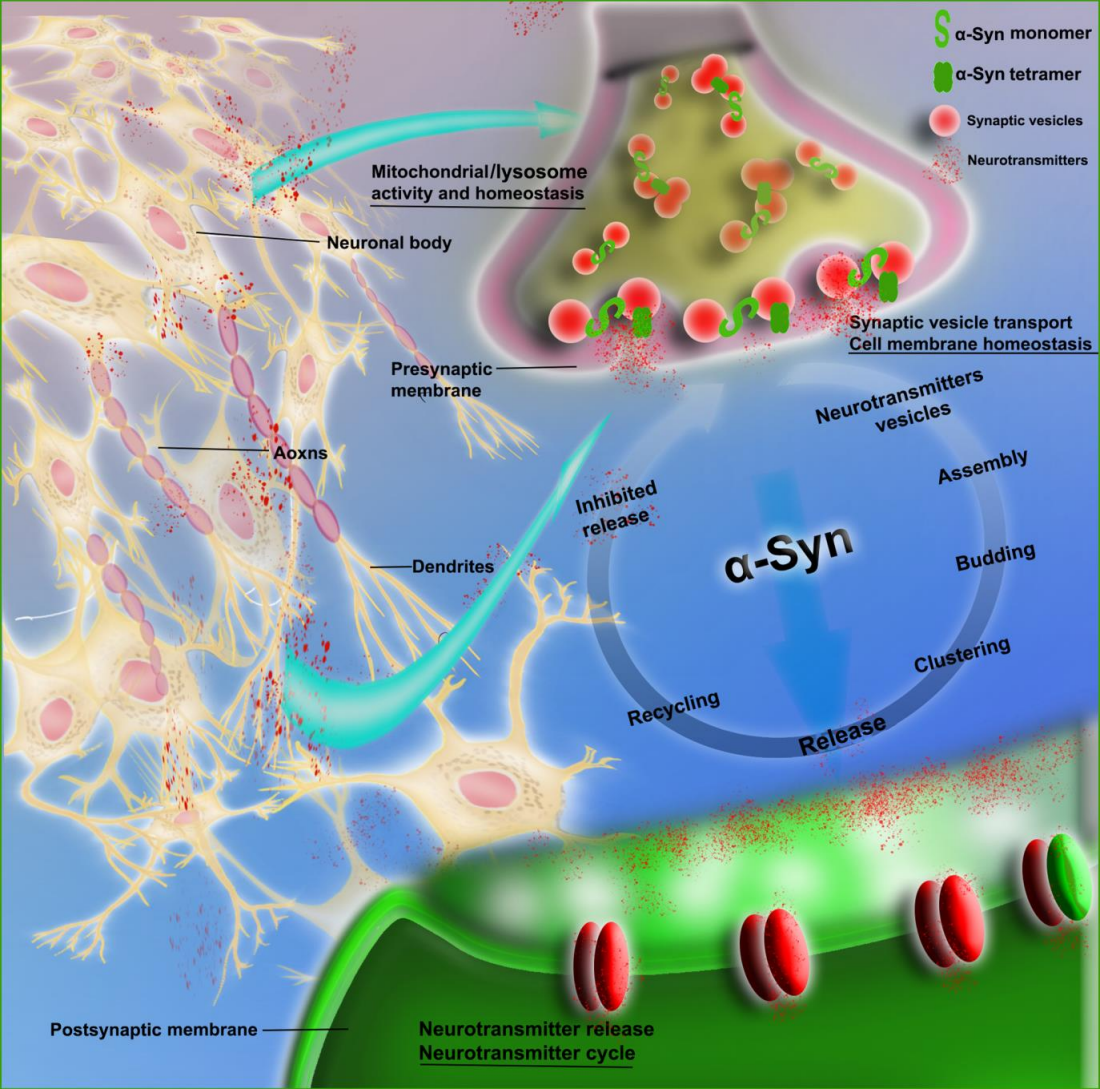
**Schematic of the physiological role of α-Syn in synaptic transmission.** The figure shows that α-Syn (monomeric and tetrameric forms) is involved in neurovesicle transport during the intricate transmission of neurotransmitters in synapses, dendrites and axons; this transport includes neurotransmitter vesicle storage, aggregation, assembly and release, as well as recovery and inhibition of neurotransmitters and other circulatory processes. α-Syn is also involved in the maintenance of cell membrane homeostasis and the normal functioning of mitochondria and lysosomes.

α-Syn participates in the dynamics of synaptic vesicle transport, promotes the interaction between synaptic vesicles, and improves their assembly, providing support for the functions of exocytosis and endocytosis, in which stability of the membrane is a key step [14]. Evidence suggests that α-Syn is involved in synaptic vesicle transport, i.e., vesicle docking, recovery and/or reaggregation. Physiologically, α-Syn acts as a soluble NSF attachment protein receptor (SNARE) chaperone, promoting the assembly of the SNARE complex to release neurotransmitters, with involvement in dopamine (DA) synthesis, transport and recovery. α-Syn maintains the stability of neurotransmitters by regulating synaptic vesicle fusion, aggregation and transport between the storage and releasable pools, as well as the interaction with neurotransmitter membrane transporters. In addition, supplementing the activity of cysteine choline protein α (CSPα) promotes synaptic integrity [15]. The selective regulation of glutamate neurotransmission alters the level of extracellular α-Syn. It is possible that the mechanism of α-Syn activity-dependent release participates in specific neural networks, maintaining precise control of the neurotransmitter cycle (Figure 2).

## α-Syn is involved in cell membrane homeostasis

α-Syn is a soluble protein that transiently fused to the membrane. Studies have shown that the balance between order and disorder in the α-Syn protein system is essential for the regulation of membrane affinity, which can induce synaptic vesicle aggregation and self-assembly into amyloid fibrils on the surface of biofilms. Of course, the more complex membrane components are not easily damaged by oligomers. At the structural level, the separation between the helical binding conformation and the disordered conformation, especially around the NAC region, seem to affect the key properties of α-Syn, such as the promotion of membrane-binding affinity or synaptic vesicles [16]. NTD acetylation to form the NTD helix can stabilize the interaction of the lipid membrane with α-Syn micelles and increase the affinity of the NTD for physiological membranes [17]. The ability of proteins to combine with lipids to promote the α-helical conformation of the NTD segment has been fully demonstrated. This prevents fibrils from forming and stabilizing physiological polymers, which together with monomers can improve the assembly of SNARE complexes and the circulation of synaptic vesicles [1, 19]. At the molecular level, the ability of α-Syn to form pores in biofilms or to interact with specific proteins in organelles and the cytoplasm may be the factor that determines the toxicity of this protein [18]. In summary, there is increasing evidence that the balance between the ordered and disordered conformations of α-Syn on the surface of biofilms is critical for membrane stability.

## α-Syn participates in mitochondrial activities

Neurons, Ranvier somatic cells, synapses, and nodes all have high energy requirements. The maintenance of mitochondrial dynamics involves multiple processes that ensure that these high energy requirements are met, namely, fusion, fission, transport, and engulfment [20], whose functions include mitigating pretouch Ca2+ levels, maintaining the membrane potential, and transport along axons and nerves. The absorption and recycling of transmitters provides energy [21]. Each of these processes is interconnected through complex relationships that maintain a functional mitochondrial network throughout the neuron life cycle. Monomeric α-Syn can increase ATP synthase efficiency and mitochondrial function [22]. At nanomolar concentrations, α-Syn reversibly blocks voltage-dependent anion channels (VDACs), which are the main channels in the outer mitochondrial membrane and control the passage of most metabolites into and out of mitochondria, protecting cells from oxidative stress [23].

## Other functions

The expression of WT α-Syn in primary brain cells also protects the cells from neurotoxicity caused by manganese [24]. Hsiao et al. reported a new role of α-Syn, highlighting its importance in neuronal cholesterol regulation, and identified novel therapeutic targets for controlling cellular cholesterol levels [25]. Eichmann et al. observed that α-Syn can form high-density lipoprotein-like (HDL-like) particles, and all human Syn proteins can form stable and homogeneous HDL-like particles with different morphologies [26].

## Pathological Characteristics and Pathogenic Mechanism of Aggregated α-Syn

The most complicated and diverse aspect of aggregated α-Syn is its pathological manifestations. Due to its structural instability and the complexity of its mutations, there is neither a precise pathological mechanism nor a clear targeted drug for this protein. Toxic α-Syn forms can negatively affect various key cellular processes, including mitochondrial function, endoplasmic reticulum (ER) stress, and protein folding, leading to cell dysfunction and death, protein degradation, and abnormal axonal transport and presynaptic function [18]. The pathogenic mechanism and pathological characteristics of α-Syn have been explored from the following perspectives.

### Characteristics of α-Syn aggregation

In disease states, α-Syn has an increased tendency to self-assemble and can form more than one type of small assembly (for example, dimers, trimers, tetramers, and larger oligomers); these assemblies have a fibril structure with a β-sheet structure, up to the size of inclusion bodies (i.e., Lewy bodies, LBs) [27]. Dimerization is the first step of α-Syn aggregation and accelerates α-Syn aggregation at acidic pH due to electrostatic effects [28]. For example, aggregates proliferate much faster in certain intracellular locations (including the ER and lysosomes) at a moderate acidic pH (6) than at a normal physiological pH [29]. In addition, point mutations promote dimerization and indicate that the structural heterogeneity of α-Syn dimers may lead to different aggregation pathways [30]. The dimerization of α-Syn accelerates the formation of fibrils. The dimer's β-hairpin region is adjacent to the nonamyloid β component (NAC) of α-Syn. Since the formation of the β-hairpin can accelerate the aggregation of α-Syn, small molecules that can bind to these regions and inhibit the process of β-hairpin formation may effectively inhibit α-Syn aggregation [31]. Furthermore, α-Syn oligomers with the same mass concentration are more effective than monomers or fibrils in clearing lipid vesicles. The conversion mechanism from soluble α-Syn monomers to disease-related oligomers, especially dimers, has become a hot topic of recent research.

PTMs of α-Syn, including phosphorylation, nitration, ubiquitination, glycosylation, and CTD truncation, are the main cause of its oligomerization [32], and there are multiple combinations of simultaneous modifications.

## Phosphorylation

A gradient of phosphorylated α-Syn accumulates in synapses (presynaptic> presynaptic+postsynaptic> postsynaptic), and phosphorylated α-Syn was found at presynaptic ends in dementia patients with LBs, mainly manifesting as small phosphorylated α-Syn aggregates, which are related to changes in synaptic morphology. Overall, pathologically phosphorylated α-Syn may disrupt the structure and function of synapses in patients with LB dementia [33]. In the brains of healthy individuals, only a small portion (4%) of the total α-Syn is phosphorylated at residue Serine-129 (Ser-129). In contrast, in Parkinson’s disease (PD) brains containing LBs, Ser-129 phosphorylation is the most common (approximately 90%) form of PTM for α-Syn [34]. In conclusion, Ser-129 phosphorylation of α-Syn can promote the accumulation of oligomeric α-Syn *in vitro* and can accelerate the formation of α-Syn inclusions.

## Nitrosation

Research suggests that Tyr-125 may promote the dimerization of α-Syn under nitrosation stress, leading to its subsequent oligomerization [35]. Oxidation and nitration of tyrosine residues in preassembled α-Syn fibers can stabilize these fibers and enhance the formation of sodium lauryl sulfate (SDS)-insoluble, thermally stable polymer aggregates, indicating that oxidative stress and nitrosyl stress are involved in its pathogenesis [36]. In addition, the intermolecular interaction between NTD and CTD of α-Syn induces α-Syn oligomerization mediated by nitrification.

## Ubiquitination

Several proteolytic systems are involved in dysfunctional degradation pathways and α-Syn aggregation, including the ubiquitin proteasome system (UPS) and the autophagy lysosomal pathway (ALP) during α-Syn degradation [37]. α-Syn aggregates colocalize with ubiquitin in an immunoreactive manner in neurons. Although this nitrosation stress is partly the result of the inflammatory changes produced by α-Syn, there is also evidence that NO causes abnormal protein accumulation by disrupting the UPS. The UPS is mainly responsible for the short-term degradation of soluble proteins, while the ALP can degrade long-standing macromolecules and cytoplasmic components, leading to functional organelle disorders. The failure of these functionally interconnected proteolytic systems may be accompanied by the accumulation of aggregated α-Syn, which will eventually interfere with normal cellular function and promote the pathogenesis of PD [38].

## Glycosylation

Glycosylation enhances α-Syn-related neurodegeneration in synucleinopathy. Glycosylation increases the oligomerization of α-Syn and interferes with the NTD of the protein, thereby reducing the capacity of α-Syn to bind to the lipid membrane. Saccharification is an inevitable age-related PTM that enhances α-Syn toxicity. Glycosylation reduces membrane binding, hinders the clearance of α-Syn, and promotes the accumulation of toxic oligomers, thereby impairing neuronal synaptic transmission [39]. Glucose is easily metabolized to produce reducing sugars, which covalently react with proteins to produce advanced glycosylation end products (AGEs), which are always responsible for protein function [40]. With age, brain defenses against glycosylation (such as glutathione, reduced glutathione and GLO1, and the glyoxalase system (GLO1)) decrease, as in the substantia nigra (SN) in PD [41]. Methylglyoxal levels increase α-Syn glycosylation and increase with age and PD [42]. In addition, glycosylation has long been known to enhance neurodegeneration in a Huntington disease model, further suggesting a link between hyperglycemia and neurodegeneration [43]. Determining the molecular mechanism by which glycosylation alters protein homeostasis and contributes to synucleosis can lead to an important breakthrough, linking aging to neurodegeneration and supporting the discovery new therapeutic targets for intervention in synucleosis. In addition, glycosylated α-Syn can impair the long-term enhancement of the hippocampus [42]. Overall, disturbances in the stable components of these glycosylated proteins can lead to the accumulation, aggregation, and cytotoxicity of α-Syn.

## CTD truncation

The conformational changes in the CTD of α-Syn in cells may be involved in the initial steps by which exogenous α-Syn aggregates form fibrils [44]. The truncated α-Syn form of CTD strongly induced the formation of LB. Intracellular repair after oxidative damage fails to target the CTD modification site of α-Syn [45]. Specific physiological CTD-truncated forms of α-Syn have significant aggregation properties, including the capacity to increase the virus-like aggregation and inoculation activities of α-Syn fibrils. In addition, CTD truncation exacerbates aggregation and α-cytotoxic synuclein, forming a vicious cycle in PD [46]. CTD-truncated fibrils show superior spread in stimulating α-Syn aggregation, and CTD α-Syn truncation in LBs is associated with cysteine protease activity, promotes amyloid formation and contributes to the pathogenesis of PD [47].

## Aggregated α-Syn and mitochondrial dysfunction

The α-Syn protein can directly form plasma membrane channels or change its activity, thereby changing the membrane's permeability to ions; this protein is also associated with mitochondrial abnormalities leading to mitochondrial dysfunction (i.e., mitochondrial depolarization, Ca^2+^ metabolic imbalance, and cytochrome C release), interference with autophagy regulation, and alteration of calcium homeostasis or mitochondrial fragmentation [48]. The interaction of α-Syn with spectrin causes pathological changes in the actin cytoskeleton and induces mitochondrial dysfunction and downstream neurotoxicity [49]. The pathogenic characteristics of α-Syn misfolding are also time-dependent pathological cascades of toxic reactions that begin with mitochondrial oxidative stress, lead to the accumulation of oxidized DA, and ultimately cause reduced glucocerebrosidase (GCase) activity, lysosomal dysfunction and α-Syn accumulation.

## Aggregated α-Syn and lysosomal dysfunction

*in vivo*, lysosomes engulf misfolded α-Syn aggregates, but incomplete lysosomal clearance mechanisms can also promote the accumulation of soluble α-Syn oligomers, which may be the key to disease progression [50]. Incorrect presynaptic peripheral α-Syn aggregation leads to lysosomal dysfunction, and this restoration of presynaptic function prevents neurodegeneration caused by lysosomal storage disease [51]. In sporadic PD, the probability of heterozygous mutations in the lysosomal hydrolase GBA1 is approximately 7%. GBA1 mutations that cause a moderate reduction in GCase by 30-50% can promote the development of PD, but the mechanism is not clear. In short, the presence of normal lysosomes and α-Syn are complementary factors that influence each other.

## Aggregated α-Syn, oxidative stress and free radical damage

Compared with surrounding organs, the brain is extremely susceptible to oxidative stress due to its extremely high polyunsaturated fat content and relatively low antioxidant activity [52]. Toxic α-Syn oligomers can affect cells in a variety of ways, including membrane destruction, mitochondrial depolarization, cytoskeletal changes, impaired protein clearance pathways, enhanced oxidative stress, and free radical damage. Much evidence shows that there is a two-way relationship between the oligomeric nature of α-Syn and the generation of reactive oxygen species (ROS) [53]. Lipid peroxidation promotes intracellular accumulation and then squeezes out toxic α-Syn as the "seed" [54]. This "seed" is then internalized by neighboring neurons, spreading the neurodegeneration process. The oxidative stress reaction destroys the ability to scavenge free radicals. Excessive free radicals can trigger the pathological production of misfolded proteins, lead to abnormal mitochondrial function, and stimulate neuronal cell apoptosis pathways [55]. The peroxidase activity of cytochrome C contributes to the formation of free radicals and α-Syn oligomerization, and α-Syn increases neuronal death by colocalizing with cytochrome C [56].

## Aggregated α-Syn and neuroinflammation

In PD and related diseases, it is important to understand the exact mechanisms of glial regulation [57], which can trigger changes in the CNS immune microenvironment, leading to outcomes such as proinflammatory responses. We propose a mechanism in which α-Syn secreted from neurons acts as a trigger that causes changes in glial cells. Studies have shown that α-Syn fibrils begin to recruit major histocompatibility complex class II (MHC II)-expressing cells in the rat brain before neurodegeneration begins; these cells consist of both resident microglia and peripheral cells, including monocytes, macrophages and lymphocytes [58]. Microglia serve as innate immune cells in the CNS. On the one hand, microglial activation is necessary to clear debris from apoptotic DA neurons. On the other hand, microglial overactivation leads to free radical damage and increases the production of cytokines and chemokines. Astrocytes have multiple functions in the CNS, from brain development to synapse formation and from blood flow barrier control to myelin sheath formation. Astrocytes can also absorb extracellular α-Syn aggregates, at a rate between those observed in neurons and microglia. Evidence for α-Syn activation of stellate cells has also been confirmed in other neurodegenerative diseases for which protein accumulation is a key pathological marker [59]. After neurons transfer α-Syn to astrocytes, astrocytes display a proinflammatory response, producing a variety of proinflammatory cytokines and chemokines. From these findings, the importance of astrocytes in the inflammatory process that progresses during synucleosis has been demonstrated.

## The Mechanism of α-Syn in Traumatic CNS Diseases

### Traumatic spinal cord injury

### Overview of spinal cord injury

Spinal cord injury (SCI) is a serious CNS trauma of the spinal cord that involves various causes of organizational structure and functional impairment and results in varying degrees of damage to sensory and motor functions, causing SCI-related autonomic dysfunction (AD) [60–61]. The disease can lead to permanent disability, with its attendant sudden autonomic function disorders such as orthostatic hypotension, autonomic reflexes, sympathetic activity surge failure and bladder, rectum, and sexual dysfunction [62]. SCI causes substantial pain to patients but is also a heavy burden for society and countries. In recent years, the incidence of SCI has shown annual increases. According to the literature, the annual incidence of SCI in Asian countries and regions is approximately 19.5-56.1 cases per million people, with 11,000 new cases of SCI in the United States per year [63]. For the first time in years, the burden of SCI was estimated at more than 900 million cases in 2016 [64]. From the perspective of the pathophysiology of SCI, the primary injury is usually attributed to local injury, which directly causes tissue defects, edema and neuronal death at the injury site; however, the more destructive lesions are secondary injuries. Many studies, including our earlier studies, have found that tissue lesions after SCI are time-dependent and spatially progressive. The damage increases with time, and more distant parts of the spinal cord are involved [65]. Typically, a series of biochemical cascade events, including activation, inflammatory mediators, oxidative stress and free radical damage, abnormal protein aggregation (including α-Syn) and glial cell activity, result in delayed neuronal death [62, 64, 66]. A secondary SCI injury can develop several months after the early stages of SCI development and is the current focus of a series of modern medical interventions.

### α-Syn-Ser-129 phosphorylation and SCI

It is unclear what causes the transformation between the physiological function and pathological aggregation tendencies of α-Syn, and this active conformational change also suggests that the balance between normal and abnormal behavior of the protein is very delicate. α-Syn-Ser-129 phosphorylation and dephosphorylation, the main PTM modes, are regulated by protein kinases and protein phosphatases, respectively. It is unclear which kinases phosphorylate Ser-129 on α-Syn in cases of disease. Polo-like kinase 2 (PLK2) is an important serine/threonine kinase that phosphorylates α-Syn at Ser-129 [67]. Neuropathological analysis of the brains of elderly nonhuman primates showed that increased expression of PLK2 was associated with increased levels of phosphorylated Ser-129-α-Syn. In addition, PLK2 was colocalized with phosphorylated Ser-129-α-Syn [68]. This finding supports the important role of PLK kinases in α-Syn phosphorylation at Ser-129 in the brain and suggests that PLK2 is responsible for this activity under physiological conditions [67]. Overexpression of PLK2 and PLK3 in DA neurons induces endogenous Ser-129 phosphorylation of α-Syn; however, its survival is not impaired, and the effect of functional phosphorylation on the interaction of α-Syn with specific protein chaperones may be significant and highly site-specific [68]. Sato et al. overexpressed G protein-coupled receptor kinase 6 (GRK-6), which increased the content of Ser-129-phosphorylated α-Syn in DA neurons and caused strong neurodegeneration and α-Syn pathological inclusions [69]. Protein phosphatase 2 (PP2A) is a ubiquitous cytoplasmic serine/threonine phosphatase that accounts for more than 50% of the serine/threonine phosphatase activity in the brain. PP2A is important for α-Syn dephosphorylation at Ser-129. Insoluble α-Syn can reduce the activity of PP2A, and overexpression of PP2A can prevent neuropathological changes in mice caused by overexpression of α-Syn. Metformin reduces Ser-129-phosphorylated α-Syn levels through mTOR-dependent PP2A activation [70]. Phosphorylation of Ser-129 under stress conditions increases the influx of extracellular Ca^2+^ and prevents the accumulation of insoluble α-Syn by causing the proteasome to perform a function complementary to that of the lysosome. However, phosphorylated Ser-129 may provide an ineffective antidegradation signal for aggregates, leading to extensive phosphorylation of the aggregates [71]. Our recent research demonstrated that α-Syn and p-α-Syn (phospho Ser-129) expression increased significantly in the early stages of SCI, and the differential expression was mainly concentrated in the white matter of the spinal cord, scattered in a punctate pattern [65, 72]. In recent years, an increasing number of studies have attached importance to this finding.

### α-Syn and SCI neuroinflammation

PTMs of α-Syn can cause the activation of microglia or monocytes and produce corresponding proinflammatory cytokines. α-Syn acts on Toll-like receptor 2 (TLR2) on microglia. TLR2 transmits the α-Syn signal that triggers the cascade, but there is also the possibility that other secondary receptor molecules participate in the internalization and clearance processes. Qiao et al. demonstrated with *in vivo* and *in vitro* experiments that neurons subjected to ischemia/reperfusion in the injured spinal cord have increased levels of α-Syn expression and release and cause microglial activation through TLR2 [73]. In WT oligo-α-Syn-pretreated rat primary microglia, TLR-2 was upregulated, and it was interesting that TLR-3 and 7 were downregulated; TLR-2 knockout (KO) inhibited the inflammatory factors TNF-α and IL-1β [74]. Furthermore, only secreted forms of α-synuclein oligomers were identified as capable of activating TLR2, while neuronal cytoplasmic α-synuclein did not activate TLR2. In addition, it has been speculated that TLR4 is also involved in α-Syn-induced microglial and astrocyte activation [75]. Furthermore, Qiao et al. demonstrated the role of α-Syn in microglial migration by isolating primary microglial cells from Sprague Dawley rats and exogenously exposing them to three different doses of SNCA oligomers [76]. Another study by Qiao et al. demonstrated that α-Syn induces microglial migration through pyruvate kinase M2-dependent glycolysis [77]. Based on qRT-PCR and western blot data, the authors also found that SNCA can increase the mRNA and protein levels of hypoxia-inducible factor-1α (HIF-1α) in microglia in a dose-dependent manner [76]. In addition, HIF-1α has been shown to be involved in microglial chemotaxis and the release of proinflammatory cytokines [77, 78]. Nitrogen-aggregated α-Syn activates primary microglia and increases the production of TNF-α, interleukin-1β (IL-1β), monocyte chemotactic protein-1 (MCP-1) and interferon-γ (IFN-γ) [79].

Our research on SCI found that α-Syn is an important promoter of neuroinflammation. Microglial cells phagocytose α-Syn accumulated in the environment and have the capacity to target light chain 3B (LC3B)+ autophagosomes for degradation [80]. In addition, microglial Fc-γ receptors (FcγR) take up extracellular accumulated α-Syn, triggering a downstream NF-κB-dependent signaling cascade (including chemokine production) [81]. Interestingly, FcγR^-/-^mice were protected from neuroinflammation and neurodegeneration after AAV-mediated α-Syn overexpression, suggesting that phagocytosed aggregated α-Syn enters microglia, which is important for inducing an immune response that causes neurodegeneration [81]. Fu H et al. suggested that complement components, especially C3 and CR3, may be related to microglial uptake of α-Syn [82].

In SCI secondary injury, silencing α-Syn can reduce the activation of microglia/astroglia, reduce the expression of iNOS, and reduce microglial toxicity via phenotypic transformation from the M1 phenotype to the protective M2 phenotype; these effects are accompanied by a significant increase in Arg-1 and IL-10 expression and regulation of neuroinflammation in the spinal cord. α-Syn-silenced rats also showed reduced IL-1β, TNF-α, and IL-2 expression in serum from peripheral blood, which significantly increased the expression of the anti-inflammatory cytokine IL-10. In addition, we also tested whether downregulating α-Syn can reduce the expression of matrix metalloproteinase-9, which may improve the function of the blood-spinal cord barrier, which is the key barrier for maintaining the stability of central and peripheral immunity [72]. Overexpression of neuronal α-Syn results in increased expression of type 1 angiotensin receptors and increased NADPH oxidase activity, as well as significant increases in the number of OX-6-positive microglia and iNOS, TNF-α, IL-1β, and IL-6 expression [83]. In addition, markers of immunomodulatory M2 microglial phenotypes, such as Arg1, have been observed to decrease significantly; however, this phenomenon was observed concomitant with concurrent use of the angiotensin type 1 blockers candesartan and telmisartan, which are inhibited by treatment [16]. Mesenchymal stem cells (MSCs) enhance α-Syn clearance via M2 microglial polarization [84]. When the immunodegradation and repair processes of M2 microglia are inhibited, M1 microglia dominate the damage site at the end of the disease; it is unknown whether these two phenotypes are compatible or contradictory, but α-Syn changes the microglial phenotype to complicate the disease. We comprehensively analyzed the effects of α-Syn knockdown on transcript levels in SCI through transcriptomics technology and found that enhancement of the cholinergic pathway may be an important way to reduce neuroinflammation. Promoting neurogenesis by reducing α-Syn at the site of SCI injury may be due to the upregulation of muscarinic cholinergic receptor subtype 2 (Chrm2) and nicotinic cholinergic receptor β2 (Chrnb2) on the cholinergic pathway [65].

### Propagation of α-Syn and SCI

α-Syn folding and assembly can produce species with different pathological effects: easy-to-extend fibrillar-like oligomers with parallel β-sheet arrangements can be used for disease and key pathogen transmission, and oligomers with antiparallel β-sheet arrangements can accumulate in cells [85]. From a micro perspective, the spread of α-Syn from cell to cell requires that α-Syn be released into the extracellular space and absorbed by recipient cells. In addition, intrinsic α-Syn requires access to the cytoplasm and/or target organelles of recipient cells. The virus-like hypothesis of α-Syn pathology proposes a method of transmitting misfolded α-Syn from one neuron to another. This hypothesis assumes that misfolded α-Syn effectively aggregates. When released and absorbed by adjacent cells, this pathological α-Syn forms a "seed", which will further misfold and aggregate. This assembly resembles a "prion", with self-replicating and self-transmitting capacities and this novel "prion" behavior may further lead to synaptic failure in synucleinopathy [86]. Transplantation of pathological α-Syn in the brain can cause rapid progressive neurodegenerative synucleinopathy in mice [87]. α-Syn spreads disease through self-modeling mechanisms similar to those of viral diseases, such as Creutzfeldt-Jakob disease [27].

From a macro perspective, the pathology of α-Syn diffuses into the brain and can deteriorate the surrounding autonomic and somatic nervous systems [87]. A major pathway of disease progression may originate in the enteric nervous system, reach the dorsal motor nucleus of the vagus nerve of the lower brainstem in a retrograde manner and travel along the brainstem to the midbrain, forebrain and cerebral cortex. The spinal cord center may be involved through the descending projection of the lower brainstem nucleus and the sympathetic projection that connects the enteric nervous system to the peripheral ganglia and preganglionic nucleus of the spinal ganglia [88]. Many synucleinopathies are accompanied by AD. A large number of α-Syn aggregates can be detected in the intestinal autonomic nerves of SCI patients, further illustrating that the transmission of α-Syn may occur in weak autonomic nerves with myelinization. Interestingly, research has found that nornicotine, a nicotine metabolite involved in saccharification, chemically modifies amyloid to prevent aggregation [89]. In summary, the development of experimental cells and animal models can help explain the mechanism by which aberrant α-Syn aggregates and the mechanism by which axonal connectivity spreads, which has facilitated the initiation of improved disease treatment strategies for potential synucleinopathy. Recently, accumulated evidence has shown that there is a close relationship between the differential expression profile of α-Syn and the selective vulnerability of certain neuronal populations.

PD and other neurodegenerative diseases associated with changes in α-Syn often cause AD, and in most cases, this synapse protein is expressed in large amounts in peripheral autonomic neurons [90]. SCI is often accompanied by AD, and its pathogenesis is unclear, but it may be related to the selective involvement of α-Syn in autonomic neurons. Perivascular nerve fibers containing α-Syn were detected in the aortas of mice, while aortic endothelial cells and muscle fibers containing the protein were not detected.α-Syn is present in the sympathetic fibers supplying the rat aorta and provides evidence that changes in the α-Syn levels in the perivascular fibers contribute to regulating vascular function [91].

### New treatment direction for SCI: target α-Syn

α-Syn has many important influences on the physiological and pathological processes of SCI due to its unique structure and its important presynaptic structure in the CNS. The overexpression or aggregation of α-Syn increase neuroinflammation after SCI. Sakurai et al. observed biomarkers in rabbits with persistent SCI and noted that motor neurons (neurons that were eventually found to be dead) were stimulated to increase α-Syn levels 8 hours after stimulation [78]. Feng et al. showed that SNCA was also downregulated to promote the expression of ciliary neurotrophic factor (CNTF), inhibit neuronal apoptosis, and promote neural regeneration [92]. Many studies have targeted the treatment of neuroinflammation induced by α-Syn. For example, methyl jasmonate, an effective antioxidant and anti-inflammatory compound, delays PD, possibly by inhibiting oxidative stress, releasing proinflammatory cytokines, and downregulating the expression of NF-κB and α-Syn [93]. Apigenin protects rat models of PD from neurodegeneration and degeneration by inhibiting neuroinflammation and oxidative stress-mediated apoptosis [94]. Lipoprotein deficiency increases the aggregation and phosphorylation of α-Syn and neuroinflammation by reducing peroxisome proliferator-activated receptor γ (PPARγ), which causes age-related loss of dopaminergic neurons and impaired motor coordination [95]. Similarly, the (molecular chaperone) cluster protein and α2-macroglobulin bind directly to the exposed hydrophobic region on the surface of the α-Syn oligomer. The combination of these two molecular chaperones reduces the capacity of the oligomer to permeate the lipid membrane and prevents oligomer-induced increases in ROS production in cultured neuronal cells [96]. Interestingly, motor neuron numbers were improved by vagus nerve stimulation [97]. In addition, knocking down α-Syn can improve neuronal survival in the cerebral cortex of SCI rats. More significantly, Jankovic et al. also studied the safety of an anti-Syn antibody (PRX002) in humans, which may prove beneficial for reducing the progression of neurological diseases that occur after acute SCI in humans [98].

In general, α-Syn is mainly involved in glial cell recruitment, activation and migration; phenotypic changes after SCI; neuroinflammation; increased expression of inflammatory mediators; changes in neurotransmitters, such as dopaminergic and cholinergic neurotransmitters; and changes in neuroprotective factors, such as CNTF. Targeting α-Syn may be of great significance in the diagnosis and treatment of SCI.

### Traumatic brain injury

Traumatic brain injury (TBI) is one of the leading causes of death among young people in developed countries. In the United States, 1.7 million traumatic events occur each year, causing 50,000 deaths. The causes of TBI include traffic accidents, falls, gunshot wounds, sports, and combat-related incidents [99]. As with SCI, the initial injuries of TBI are caused by mechanical factors that damage neural tissue. From animal experiments to clinical studies, secondary lesions appear a few minutes after trauma and can develop for months or even years. Cell and molecular events lead to the secondary events of brain cell death and neurodegeneration, which are accompanied by a high risk of certain neurodegenerative diseases [100]. Cognitive impairment becomes a major sequela of TBI in rodent models or patients with secondary injury [101]. Another study showed histological evidence of chronic traumatic encephalopathy (CTE) in neurodegenerative diseases, with PD accounting for 16% of cases [102]. The biological mechanisms that connect brain trauma and neurodegenerative diseases require further study. Studies on the correlation between chronic TBI and other neurodegenerative diseases have shown that repetitive TBI promotes the accumulation of abnormal aggregate proteins, including TAR DNA-binding protein 43, amyloid beta protein and α-Syn. Increased α-Syn in the CSF was observed in patients with severe TBI (p = 0.0008). A large increase in α-Syn in the CSF may indicate widespread neurodegenerative changes and reflect secondary neuropathological events that occur after injury [103]. Similarly, in a study of the feasibility of α-Syn as an objective biomarker for the diagnosis and prognosis of mild TBI, it was believed that amyloid-β (Aβ) peptide, tau protein and α-Syn are involved in the downstream events of the TBI-induced idiopathic cascade [104]. In patients with severe TBI, it is often difficult to predict survival or long-term outcomes, especially in the first few days after injury, but the α-Syn levels in patients in the first 24 hours after injury were significantly higher than the levels in the control group. In patients who survived the injury, the α-Syn levels tended to be normal after 3 days, while in patients who did not survive, the α-Syn levels in the CSF remained increased until 8 days after the injury [105]. Therefore, the measurement of CSF α-Syn may be a valuable prognostic marker.

Brain trauma leads to the development of PD-related pathology in mice. Interestingly, compared with astrocytes, microglia accumulate a large amount of α-Syn. In Alzheimer’s disease and PD mouse models, bone marrow MSCs, macrophages and microglia were delivered to the brain via the nasal cavity, showing an intracellular amyloid beta (APP/PS1 model) or α-Syn (Thy1-h[A30P]α-Syn model) immune response [106]. In addition, it has been reported that α-Syn aggregation may cause dopaminergic neuron loss or synuclein pathological changes after TBI. These changes occur in the environment of vascular fragility and microglial activation shortly after TBI. Loss of dopaminergic neurons is often accompanied by vascular and immunological changes. IgG exudation was observed in injured rats within 1-2 days, while no IgG exudation was observed in control rats at 7 or 28 days, indicating that blood-brain barrier (BBB) permeability had temporarily increased [107]. Some studies have shown that in astrocytes, α-Syn-induced proinflammatory responses occur in a TLR4-dependent manner [59]. However, unlike inflammatory signals, the uptake of α-Syn by astrocytes is independent of TLR4. *in vitro* studies have shown that the neuroprotective effect of anti-TLR2 antibodies is mediated by preventing α-Syn transmission from neurons to neurons and from neurons to astrocytes; otherwise, the antibodies will promote an NF-κB-dependent proinflammatory response [108]. For example, in the multiple system atrophy model, the activation of astrocytes is directly related to the proximity of α-Syn inclusions in oligodendrocytes, called glial cytoplasmic inclusions [109].

α-Syn stimulates astrocytes; the potential roles of neuroinflammation and neuroprotection and how to establish and maintain an inflammatory microenvironment with this protein are still unknown. Neuronal α-Syn can be directly transferred to astrocytes through sequential exocytosis and endocytosis and induces astrocytes to produce an inflammatory response. Early in the period after craniocerebral injury, the rat TBI model had vascular abnormalities and inappropriate microglial activation, and the mouse model had dopaminergic neuron loss due to α-Syn and increased microglial activity. The TBI-related changes in α-Syn depend on age and the time after the head injury occurred. For example, after 60 days of TBI in rats, brain tissue was collected and found to be colocalized with α-Syn, tyrosine hydroxylase (TH), an enzyme that synthesizes DA neurons, and the major histocompatibility MHC II. Compared to the control neurons, surviving dopaminergic neurons had significantly reduced TH-positive expression in the dense substantia nigra (SNpc), and the α-Syn accumulation detected in the ipsilateral SN was increased [110]. α-Syn is a pathological link between the chronic effects of TBI and the symptoms of PD, as evidenced by the significant overexpression and abnormal accumulation of α-Syn in the inflammatory SN exposed to chronic TBI [110]. Although microglial and subsequent astrocyte activation is often considered a secondary response to neuronal damage, proinflammatory changes within microglial cells have been observed to cause evidence of injury to DA neurons in PD animal models [109]. There is a difference between the location of neuronal death caused by α-Syn and the differential expression of brain-dependent α-Syn. Expression patterns of α-Syn are different between excitatory and inhibitory hippocampal neurons; α-Syn is highly expressed in neuronal cell bodies in certain early brain regions affected by PD, such as the olfactory bulb, the dorsal motor nucleus of the vagus nerve, and the dense SN. The synaptic expression of α-Syn is mainly accompanied by the expression of the excitatory presynaptic marker vesicle glutamate transporter-1 (VGT-1). In contrast, the capacity of γ-aminobutyrate (GABA) to inhibit the expression of α-Syn in synapses differs among brain regions. α-Syn is clearly expressed in inhibitory synapses in the outer plexiform layer of the olfactory bulb, pale bulb, and SN reticulum but is not expressed in the cerebral cortex, subthalamic nucleus, or thalamus [111]. Similarly, the synaptic expression of α-Syn is mainly accompanied by the expression of VGT-1, an excitatory synaptic marker protein. In contrast, the inhibition of α-Syn expression in synapses differs among brain regions [112]. Another report noted that the diffuse α-Syn proximity connection assay had significantly more signals in the patient group than in the control group, including in the cingulate cortex (1.6-fold increase) and medulla reticulum (6.5-fold increase) [113].

Toxic α-Syn spreads between cells through exosomes and induces apoptosis, and microglia and astrocytes may act as regulators of α-Syn exosome delivery. Exosomes are small vesicles that are released from cells into the extracellular space. The accumulation of α-Syn at the presynaptic end affects several steps of neurotransmitter release. First, high levels of α-Syn change the size of the synaptic vesicle pool and impair its transport, forming exosomes/EVs for distribution to the extracellular space. Second, overexpression of α-Syn may relax or redistribute the protein of the presynaptic SNARE complex, leading to insufficient storage, docking, guidance, and fusion of synaptic vesicles in the active functional area. Third, α-Syn inclusions are found in the presynaptic active area, accompanied by a decrease in protein levels in the active area. In addition, during the recovery of vesicles, overexpression of α-Syn reduces the endocytosis of synaptic vesicle membranes, further impairing the exocytosis of neurotransmitters, which can trigger synaptic dysfunction and impaired neuronal communication [114]. Under physiological conditions, the ALP contributes to the intracellular homeostasis of the cytosolic protein SNCA/α-Syn. Endosome/lysosomal function is an endogenous disorder that causes pathology of α-Syn inclusion bodies. In TBI, α-Syn exosomes/EVs may lower the threshold of pathological induction/diffusion [115]. Inhibition of the ALP increased the proportion of extracellular SNCA, and ultrastructural analysis showed extensively fused polycystic autophagic cells. CSF exosomes/EVs transfer SNCA from one cell to another *in vivo*.

Mechanical stress increases brain Aβ, tau, and α-Syn concentrations in WT mice. The dose-dependent and cumulative effects of repeated mild TBI-induced mechanical stress can trigger and/or accelerate neurodegeneration by causing protein concentrations to exceed disease thresholds [116]. Because axonal damage is related to axonal transport disorders, studies have found that α-Syn accumulation often occurs in the white matter. α-Syn immunoreactivity was found in axonal dystrophy, and axon swelling was found in acute TBI [117]. In addition, a large accumulation of α-Syn was found in swollen axons, and tau protein also accumulated in both axons and neuronal cell bodies [118]. Starting one month after brain infiltration, tau in the prefrontal cortex (PFC) and hippocampus selectively increased for three months, while α-Syn and Hippo increased briefly [119]. In a study by Urya et al., a transient increase in the immunoreactivity of α-Syn in axons that traverse the striatum was observed in adult animals [118]. This accumulation of α-Syn may be the result of abnormal axonal transport and the subsequent accumulation of various proteins at the injured site. In addition, there may be a link between TBI and the oxidative/nitrative stress that produces α-Syn pathology. Impaired neurotransmission has been reported within weeks after experimental TBI, which may also be the cause of behavioral dysfunction. After TBI, the formation of SNARE complexes and the abundance of various SNARE proteins (including CSPα) were reduced [101, 120]. The soluble SNARE complex is a highly conserved mechanism that promotes vesicle docking and fusion, which is essential for neurotransmission. Overall, these results indicate that abnormal aggregation of multiple proteins occurs in swollen axons after neurodegeneration but that different proteins undergo selective regional aggregation, which may be closely related to functional neuron types and neurotransmitter transmission.

## The Mechanism of α-Syn in Vascular CNS Diseases - *Ischemic Stroke*

Cerebral ischemia triggers a complex series of biochemical and molecular events that promote neuronal death and neurological dysfunction. These include but are not limited to excitotoxicity, ion imbalance, edema, oxidative stress, ER stress, and inflammation. After cerebral infarction, a large amount of free radicals and ROS are generated, which promotes the peroxidation of cell membrane lipids and aggravates microcirculation disorder in the ischemic area. The standard treatment for acute cerebral infarction is to improve blood circulation in the cerebral ischemic area as early as possible, improve brain metabolism, and promote the recovery of nerve function. Mitochondria are the main source of ROS (90%), and increased ROS levels due to mitochondrial dysfunction can damage neuronal survival [121]. Neurons in the ischemic penumbra region after cerebral infarction also bear higher loads of ROS and reactive nitrogen species (RNS).

α-Syn is thought to be a chronic neurodegeneration-related protein that can also mediate secondary brain damage after acute brain damage. Neuronal damage due to chronic diseases (such as PD) can continue for years, while that due to acute diseases (such as stroke) continues for hours to days. Stroke can also initiate or accelerate progressive neurodegenerative processes and is a known epidemiological risk factor for AD [122]. α-Syn is mainly distributed in neurons and is permeable to the serosa. In the peripheral blood, a study that detected α-Syn in RBCs suggested that ischemia-reperfusion in stroke patients may destroy cells in the stroke area and make it easier for α-Syn to diffuse into the peripheral blood, which will cause ischemia. The content of α-Syn in the peripheral blood was the highest in the stroke group. The number of α-Syn oligomeric forms in the ischemic stroke group was much higher than that in the PD group, and it has also been revealed that the pathogenesis of ischemic stroke may be correlated to the pathogenesis of PD [123]. In the CNS, Hu et al. showed that hypoxia/ischemia increased the α-Syn protein levels in the rat cerebral cortex [124]. Subsequently, Yoon et al. reported that hippocampal CA1 neuronal loss was more pronounced in adult gerbils than in older gerbils 4 days after ischemia/reperfusion. The α-Syn level in the CA1 region of aged gerbils is higher than that in the CA1 region of adult gerbils, and this result may be related to the early induction of ROS. More importantly, the increase in α-Syn expression and neuronal death can be reduced by treatment with the antioxidant enzyme SOD1, suggesting that ROS promote the expression and aggregation of α-Syn [125]. In addition, different studies have linked the function of α-Syn to mitochondrial fusion, fission, transport, and maintenance.

After cerebral ischemia, the cellular environment, including inflammation, oxidative stress and ER stress, may provide the best conditions for α-Syn aggregation. *in vitro* studies have shown that oxidative/nitrative stress and acidity induce α-Syn oligomerization. These conditions that favor α-Syn fibrosis exist in the ischemic brain and can be used as an *in vivo* model for studying α-Syn aggregation. Middle cerebral artery occlusion (MCAO) in C57BL/6 (Thy1)-h[A30P]α-Syn transgenic mice, which significantly enhances 3-nitrotyrosine immunoreactivity at the congestion site, indicates that nitrification stress may be one of the mechanisms that mediate aggregation toxicity [126]. Therefore, the increased vulnerability of transgenic mice to ischemia suggests that α-Syn aggregates not only form during ischemia but also negatively affect neuron survival, supporting the view that α-Syn misfolding may be neurotoxic [126]. Interestingly, the use of telmisartan reduced progressive oxidative stress and phosphorylated α-Syn accumulation in stroke-resistant spontaneously hypertensive rats (SHR-SRs) after transient MCAO. Hypertension that persists after tMCAO in SHR-SRs causes long-term oxidative stress, which accelerates the accumulation of p-α-Syn. Telmisartan, a receptor activated by PPARγ, reduces this oxidative stress and the accumulation of p-α-Syn [127]. In addition, Kim et al. found that miR-7 mimic therapy can significantly reduce the induction of α-Syn after ischemia. MiR-7a-5p improves ischemic brain injury by inhibiting α-Syn, and reducing α-Syn can induce mitochondrial fragmentation, oxidative stress and autophagy, thereby reducing neuronal cell death [128].

In addition, global cerebral ischemia in α-Syn KO mice resulted in elevated prostaglandin levels, suggesting that α-Syn may aggravate in mediating postischemic inflammation. Ahmad et al. also demonstrated that stimulation of the prostaglandin D2 receptor DP1 has neuroprotective effects in the ischemic brain in rodents [129]. Another study showed that MCAO-induced transient focal ischemia in adult rats significantly upregulated α-Syn mRNA and protein levels, while knockdown of α-Syn reduced infarction and promoted better neurological recovery. α-Syn KO also weakened known ischemic pathological markers, including markers of mitochondrial dysfunction (dynamin-related protein 1, Drp1), apoptosis (cleaved caspase-3), autophagy (LC-3 II/I ratio), and oxidative stress (3-nitrotyrosine). In addition, the α-Syn protein is oligomerized, aggregated and translocated to the nucleus of neurons after stroke in rats and humans [122]. Adult rats suffered moderate ischemia-reperfusion injury, leading to α-Syn induction and phosphorylation and nuclear translocation of α-Syn and p-Ser-129, resulting in α-Syn aggregates; in addition, 4 months later, the animals formed aggregates of proteinase K [130]. After toxic injury, α-Syn translocates into the neuronal nucleus and forms a complex with histones, leading to a reduction in histone acetylation. α-Syn overexpression is thought to increase Drp1 translocation and alter mitochondrial morphology through extracellular signal-regulated kinases [131]. In addition, α-Syn forms an oligomer with a ring-like, porous structure in the membrane, which has high permeability to calcium, leading to caspase activation and death of apoptotic neurons. Overexpression of WT α-Syn caused impaired macroautophagy through Rab1a inhibition [132]. In conclusion, α-Syn is a potential therapeutic target for reducing brain damage after stroke.

## Summary and Future Prospects

We separately summarized different aspects of traumatic CNS disease and stroke, from the structural and physiological features of α-Syn to its pathological processes and disease outcomes. The structural characteristics and membrane positioning of the amphiphilic and hydrophobic α-Syn monomer control its stability in the membrane, transmembrane transport of substances, and transmission of neurotransmitters. When the body is damaged, α-Syn aggregates abnormally, causing mitochondrial dysfunction, abnormal lysosomal function, exacerbated oxidative stress, free radical damage, and a series of inflammatory cascades. Eventually, related neuronal death results in abnormal or restricted neurotransmission (dopaminergic and cholinergic neurons).

Based on this, there are still many unresolved questions about the effects of α-Syn on traumatic and vascular CNS diseases:

1). Based on the existing research, we are aware of the neurotoxic effects of α-Syn after injury, but what is the physiological effect of α-Syn on injury?

2). CNS injury is accompanied by neuroinflammation and α-Syn aggregation or upregulation. Does inflammation promote α-Syn aggregation or does α-Syn aggregation promote inflammation? Research has revealed that α-Syn is not unique to neurons but is also localized in glial cells (such as microglia, astrocytes and oligodendrocytes). However, what kind of glial cells does α-Syn affect during the secondary inflammatory response to CNS injury?

3). α-Syn is not only the most abundant protein in the CNS but also exists in the peripheral blood and peripheral nervous system. However, many studies have found that the expression of α-Syn in the peripheral blood or CSF increases and acts as a biomarker for diseases (such as SCI, TBI, and stroke). The most notable feature of traumatic CNS disease is the BBB, and the cerebrospinal barrier is damaged. Does this occur because α-Syn migrates to the peripheral blood or because local α-Syn monomers accumulate?

4). The mechanism by which α-Syn is transmitted between cells is still unknown, and whether transmission is related to the vagus nerve currently remains unclear.

First, an injury related to trauma or stroke is a serious blow to the CNS that triggers a series of abnormal pathological reactions. The overexpression and aggregation of α-Syn after injury is likely to cause the death of related neurons, such as autonomic neurons and residual neurons at the edge of the injury. It may be difficult for α-Syn to perform its physiological functions due to environmental damage. In early undamaged cells, α-Syn still performs physiological functions such as transmembrane transport, membrane stability, and precise release of neurotransmitters. The inflammatory response is the main change during the acute phase of injury. It occurs immediately after the injury. At this time, α-Syn aggregation or overexpression is very low or almost negligible; the inflammatory response decreases over time, but many studies have found that during the chronic phase of the disease, a large amount of α-Syn aggregation occurs in old age, so it is closely related to neurodegenerative diseases. Therefore, α-Syn aggregation showed a time-dependent increase after injury. In existing studies, α-Syn was found in microglia, astrocytes and oligodendrocytes. Clinically, due to ethical issues related to the use of patients' blood, an increase in α-Syn was detected in the cerebrospinal fluid. In animal experiments, α-Syn overexpression was found in a substantial portion of the injured area. We believe that the source of α-Syn may be related to the course of the disease, that it is difficult to determine the main source, and that central and peripheral blood sources may exist at the same time. From a macro perspective, we currently believe that α-Syn is likely to propagate along the autonomic nervous system because this type of nerve is unmyelinated, the axons are thin and wide, and the cell body has a high energy load. Since this remains an unexplored question, the answer is definitely complicated.
